# Idiopathic pulmonary fibrosis and cancer: do they really look similar?

**DOI:** 10.1186/s12916-015-0478-1

**Published:** 2015-09-24

**Authors:** Carlo Vancheri

**Affiliations:** Regional Centre for Interstitial and Rare Lung Diseases, Department of Clinical and Experimental Medicine, University of Catania, Via S. Sofia 78 – building 4, first floor, 95123 Catania, Italy

**Keywords:** Cancer, Idiopathic pulmonary fibrosis, Pathogenesis, Signalling transduction pathways

## Abstract

**Background:**

The aim of this opinion article is to understand to what extent idiopathic pulmonary fibrosis (IPF) can be considered, in its clinical and pathogenic features, similar to cancer. Indeed, IPF has common risk factors with cancer, a low survival, and, most importantly, epigenetic and genetic alterations, abnormal expression of microRNAs, cellular and molecular aberrances, and the activation of similar signalling pathways.

**Discussion:**

The pathogenic link between the two diseases may have a number of practical consequences. It may improve our understanding of IPF drawing on cancer biology knowledge. In addition, the recognition of similar pathogenic pathways may also encourage the use of cancer drugs for the treatment of IPF. Nintedanib, an inhibitor of tyrosine kinase receptors initially developed for cancer, has been recently approved for the treatment of IPF thanks to the observation that these receptors are also abnormally activated in IPF.

**Summary:**

The vision of IPF as a cancer-like disease may improve our understanding of the pathogenesis of this disease also opening new scenarios for repositioning cancer drugs for IPF. In addition, it may increase the level of awareness towards this dreadful disease at the public, political, and healthcare level.

## Background

According to the current pathogenic hypothesis, idiopathic pulmonary fibrosis (IPF) is the result of chronic damage of the alveolar epithelium leading to a series of events responsible for abnormal tissue repair and the profound changes of the alveolar structure that characterize this disease [1]. This altered “wound healing” process is driven by a variety of pathogenic events commonly described in other degenerative/fibrotic diseases and interestingly also in cancer. Cancer, defined by some authors as a “wound that does not heal” [2–4] has an often unknown aetiology, risk factors similar to IPF, and the presence of a specific genetic background considered important for the occurrence of the disease. Similarly to cancer, IPF affects susceptible individuals and shares common risk factors with cancer such as smoking, environmental or professional exposure, viral infections, and chronic tissue injury [1]. Based on these similarities, as well as its poor response to medical treatment and its prognosis, often worse than many cancers [5, 6], IPF has been frequently, although vaguely, compared to a type of malignant disease. It is our opinion that, apart from this obvious and circumstantial evidence, the two diseases have an incredible number of pathogenic mechanisms in common that make IPF very similar to cancer. Epigenetic and genetic alterations, abnormal expression of microRNAs (miRNAs), cellular and molecular aberrances such as the altered response to regulatory signals, delayed apoptosis, or reduced cell-to-cell communication, along with the activation of specific signalling transduction pathways are all features that characterizes the pathogenesis of both IPF and cancer [5, 6]. The presence in IPF of risk factors, genetic alterations and, in general, pathogenic pathways similar to cancer may explain the relative frequency of the association between IPF and lung cancer. In these cases, lung cancer and IPF show a similar anatomic distribution, with tumours typically being sub-pleural or peripheral and close to or within honeycomb and fibrotic areas. Further, the development of lung cancer in patients with IPF is responsible for a significantly worse prognosis; in a recent study, survival in patients with lung cancer and IPF was significantly shorter than in patients with IPF without lung cancer (median survival, 38.7 vs. 63.9 months) [7].

However, the study of the association between IPF and lung cancer with the relative clinical and therapeutic implications is far beyond the objectives of this opinion article. The aim of this article is instead to go through the scientific reasons that may support the intriguing vision of IPF as a disease with a cancer-like nature and to understand if this point of view may have positive consequences on the overall management of IPF.

## Discussion

### Epigenetic and genetic alterations in cancer and IPF

Aging, cigarette smoke, diet, and chemical and physical factors, through the methylation of suppressor genes or the hypomethylation of oncogenes, may affect the activity of some genes without changing their DNA sequences. It is recognized that these “epigenetic” changes are involved in the initiation and progression of cancer. For instance, the reduced expression of the Thy-1 glycoprotein, due to the hypermethylation of the promoter region of the gene coding for this glycoprotein, is associated with a more invasive behaviour of cancer. Similarly, the loss of expression of the same glycoprotein in IPF is related to the differentiation of fibroblasts into myofibroblasts within “fibroblast foci” [8, 9]. More recently, Rabinovich et al. [10] showed that alterations of the global methylation pattern in IPF are similar, to a certain extent, to lung cancer. Cancer is also marked by a number of genetic alterations such as micro-satellite instability, loss of gene heterozygosity, p53 and fragile histidine triad mutations, as well as telomere shortening and telomerase impairment [11–13]. These alterations, which affect a series of crucial cellular mechanisms such as cell proliferation, differentiation, senescence, and apoptosis, are also present in IPF, where a lower expression of two subunits of telomerase (h-TERT and h-TERC) and a subsequent shortening of telomeres have also been described [14–17]. Recently, it has been shown that the aberrant expression of miRNAs is also linked to initiation and progression of cancer. MiRNAs are non-coding small RNAs regulating different molecular and cellular processes through a post-transcriptional mechanism. The increased expression or inhibition of a single miRNA may control different oncogenes, suggesting that replacement or inhibition of miRNAs may be a new therapeutic target for cancer. In lungs of mice treated with bleomycin and in lungs of patients with IPF, a large variety of miRNA alterations have been described. MiR-21 that normally promotes TGF-β signalling is up-regulated, on the contrary, the increased expression of miR-155 leads to the down-regulation of a number of target genes mediating fibroblast migration. There is also evidence that miRNAs expression in IPF correlates with disease severity as demonstrated by the findings of increased levels of specific miRNAs (miR-302c, miR-423, miR-210, miR-376c, and miR-185) in lung biopsies of rapidly progressing patients with IPF [18–23]. Pandit et al. [23] showed that TGF-β, through the down-regulation of the expression of let-7d, induces the expression of the mesenchymal markers in epithelial cell lines, thus promoting the epithelial-mesenchymal transition (EMT). Furthermore, the use of an “antagomir” specific for the let-7 family results in the down-regulation of epithelial markers suggesting an important role for the let-7 family in lung fibrosis as well as a potential therapeutic approach for IPF.

### Altered cell-to-cell communication and uncontrolled cell proliferation in cancer and IPF

Tissue homeostasis is maintained by cell-to-cell communication through junctional channels named connexins that facilitate the interaction and coordination of cell functions. Cancer cells are instead characterized by an impairment of intercellular communication suggesting that, in order to proliferate, these cells need to isolate themselves from the influence of surrounding normal cells. Lower levels of connexin 43 (Cx43) have been described in many cancers, including lung and gastric cancer. Similarly, IPF myofibroblasts, have a reduced ability to express connexin 43, suggesting that the loss of proliferative control in IPF cells could be caused by an altered fibroblast-to-fibroblast communication caused by a reduced expression of connexin 43 [24–27]. The origin of myofibroblasts is very similar in both IPF and cancer. They may arise either from resident fibroblasts or from resident epithelial cells through EMT. In this case epithelial cells progressively reorganize their cytoskeleton and assume mesenchymal markers like fibronectin and alfa-smooth-muscle actin. In IPF, epithelial cells surrounding fibroblast foci express both epithelial and mesenchymal markers, suggesting that EMT is undergoing in those areas of lung tissue. These and other similar observations support an active role for EMT in contributing to lung fibrogenesis. Even though the EMT process cannot be viewed as a “cancerogenetic” event, but at most as a form of metaplasia, it is commonly considered a crucial feature of many cancers involved in early steps of carcinogenesis and cancer cell invasion [28–30]. Myofibroblasts may also derive from circulating fibrocytes originated from the bone marrow [31–33]. Regardless of their origin, cancer-associated myofibroblasts produce growth factors such as TGF-β to support their own growth and metalloproteinases, which, by destroying the extracellular matrix, facilitate the infiltration of cancer cells into the surrounding tissues. Cancer progression is also facilitated by the expression and the activity of molecules associated with delayed cell apoptosis, cancer progression, and tissue infiltration such as laminin, heat shock proteins, and fascin. It is interesting to note that, in IPF, myofibroblasts also sustain their own growth producing TGF-β which in turn stimulates their differentiation, proliferation, and collagen production. Moreover, epithelial cells surrounding fibroblasts foci also express fascin, heat shock protein 27, and laminin suggesting, once again, an important similarity between IPF cells and cancer cells [34–37].

### Activation of signal transduction pathways in cancer and IPF

A large variety of signal transduction pathways are involved in the pathogenesis of cancer and IPF. The Wnt/β-catenin signalling pathway regulates the expression of molecules involved in cancer progression and tissue infiltration such as matrilysin, laminin, and cyclin-D1. This pathway is also strongly activated in IPF lung tissue as evidenced by extensive nuclear accumulation of β-catenin at different involved sites such as bronchiolar lesions, damaged alveolar structures, and fibroblast foci [38, 39]. The PI3K/AKT signalling pathway, strongly involved in the pathogenesis of cancer, regulates cell growth, proliferation, and cell protection from apoptosis. Recently, the expression of class I PI3K p110 isoforms in IPF lung tissue as well as in tissue-derived fibroblast was assessed [40–43]. The expression of p110γ was enhanced in both IPF lung homogenates and *ex vivo* fibroblast cell lines. Furthermore, both p110γ pharmacological inhibition and gene silencing were able to significantly inhibit proliferation rate as well as a-SMA expression in IPF fibroblasts. This suggests that PI3K p110γ isoform may have an important role in the pathogenesis of IPF and can be a specific pharmacological target. More recently, researchers have focused on another signal transduction pathway frequently altered in many cancers: the JAK-STAT pathway [44]. This pathway is regulated by a family of proteins called “suppressor of cytokine signalling proteins”. According to Bao et al. [44], a lower expression of SOCS1 was identified in IPF patients and this finding has been related to severe manifestations of disease and to a worse prognosis. Another key signalling pathway strongly activated in many cancers but also in IPF is the tyrosine kinase pathway. This pathway, once activated, regulates many cell functions, such as cell growth, differentiation, adhesion, motility, and regulation of cell death, which may be associated with initiation or progression of cancer. Further, the activity of these receptors has been related to wound healing and fibrogenesis [45, 46]. The anti-fibrotic profile of multiple inhibitors of tyrosine kinase receptors has been evaluated on fibroblasts both *in vitro* and *in vivo* in a rat model of bleomycin-induced fibrosis [47, 48]. The contemporary inhibition of PDGFR, VEGFR, and FGFR resulted in significant attenuation of fibrosis even when the inhibitory drug was administered 10 days after intra-tracheal instillation of bleomycin, suggesting a novel therapeutic approach for the treatment of IPF. During the last few years, tyrosine kinase receptor inhibitors such as nintedanib, developed and already used for the treatment of non-small cell lung carcinoma and other cancers, have been tested in phase II and III clinical trials for the treatment of IPF [48–52]. Nintedanib has shown to significantly reduce the decline in lung function of IPF patients. Based on this result, this drug was recently approved as a new therapeutic option together with pirfenidone in patients with IPF.

### Summary and final considerations

According to the long list of pathogenic analogies between cancer and IPF, we might easily conclude that these two diseases are indeed similar. However, there are at least three arguments, apparently against this concept, that still need to be clarified: (1) myofibroblasts within fibroblast foci are polyclonal whereas cancer cells are thought to be monoclonal; (2) IPF is limited to the lungs, cancer metastasizes; and (3) IPF is by definition bilateral, cancer is always unilateral. These arguments, in our opinion, are largely based on general beliefs and do not weaken in any way the concept of the cancer-like character of IPF. Effectively, a number of studies have unequivocally shown that only some cancers are monoclonal whereas many others are instead marked by the same cytogenetic heterogeneity observed in IPF cells. Similarly to IPF, myofibroblastic cancers, such as desmoid tumours, are locally aggressive but unable to metastasize and ultimately 2–6 % of cancers have a bilateral and synchronous involvement [53–58].

Having removed any conceptual obstacle to the view of IPF as a disease with a close link to cancer biology, this commentary will briefly underline the main reasons explaining why this vision may be relevant not just conceptually but also clinically. The awareness of cancer as a severe and potentially deadly disease is widespread in public opinion, leading to fund-raising campaigns and gatherings of private and governmental funds, resulting in the continuous progression of basic and clinical research. The development of dedicated diagnostic tools, the realization of large screening programs, the launch of a variety of new anti-cancer drugs, as well as the creation of personalized treatments have been achieved due to the fight against cancer during the last few decades. On the contrary, the awareness of IPF as a fatal disease, with a prognosis worse than the majority of cancers, is still limited to few experts and to those patients and families who are directly involved with the disease. The concept of IPF as a cancer-like disorder of the lung may help in meeting the urgent need for a better understanding of the disease and may increase the attention given to IPF at a public, political, and healthcare level. In addition, the existence of pathogenic links between IPF and cancer may improve our understanding of the pathogenesis of IPF, taking advantage of the large knowledge that already exists of cancer biology or even “borrowing” drugs, as in the case of nintedanib, which have been developed or used for cancer. Finally, the recognition of new common pathogenic mechanisms between IPF and cancer may also encourage new IPF clinical trials with other common oncologic drugs, alone or in combination with other drugs, or even through personalized treatments as largely experimented in cancer.

## Abbreviations

Cx43: Connexin 43
EMT: Epithelial-mesenchymal transition
IPF: Idiopathic pulmonary fibrosis
miRNAs: microRNAs
